# Why and how to use the SeqCode

**DOI:** 10.1002/mlf2.12092

**Published:** 2024-02-07

**Authors:** William B. Whitman, Maria Chuvochina, Brian P. Hedlund, Konstantinos T. Konstantinidis, Marike Palmer, Luis M. Rodriguez‐R, Iain Sutcliffe, Fengping Wang

**Affiliations:** ^1^ Department of Microbiology University of Georgia Athens Georgia USA; ^2^ School of Chemistry and Molecular Biosciences, Australian Centre for Ecogenomics The University of Queensland St Lucia Australia; ^3^ University of Nevada Las Vegas Las Vegas Nevada USA; ^4^ School of Civil and Environmental Engineering, and School of Biological Sciences, Georgia Institute of Technology Atlanta Georgia USA; ^5^ Department of Microbiology University of Manitoba Winnipeg Manitoba Canada; ^6^ School of Life Sciences University of Nevada Las Vegas Las Vegas Nevada USA; ^7^ Department of Microbiology and Digital Science Center (DiSC) University of Innsbruck Innsbruck Austria; ^8^ Faculty of Health & Life Sciences Northumbria University Newcastle upon Tyne UK; ^9^ School of Oceanography, International Center for Deep Life Investigation Shanghai Jiao Tong University Shanghai China

**Keywords:** genome sequences, metagenome‐assembled genomes, nomenclature, SeqCode

## Abstract

The SeqCode, formally called the Code of Nomenclature of Prokaryotes Described from Sequence Data, is a new code of nomenclature in which genome sequences are the nomenclatural types for the names of prokaryotic species. While similar to the International Code of Nomenclature of Prokaryotes (ICNP) in structure and rules of priority, it does not require the deposition of type strains in international culture collections. Thus, it allows for the formation of permanent names for uncultured prokaryotes whose nearly complete genome sequences have been obtained directly from environmental DNA as well as other prokaryotes that cannot be deposited in culture collections. Because the diversity of uncultured prokaryotes greatly exceeds that of readily culturable prokaryotes, the SeqCode is the only code suitable for naming the majority of prokaryotic species. The start date of the SeqCode was January 1, 2022, and the online Registry (https://seqco.de/) was created to ensure valid publication of names. The SeqCode recognizes all names validly published under the ICNP before 2022. After that date, names validly published under the SeqCode compete with ICNP names for priority. As a result, species can have only one name, either from the SeqCode or ICNP, enabling effective communication and the creation of unified taxonomies of uncultured and cultured prokaryotes. The SeqCode is administered by the SeqCode Committee, which is comprised of the SeqCode Community and elected administrative components. Anyone with an interest in the systematics of prokaryotes is encouraged to join the SeqCode Community and participate in the development of this resource.

## INTRODUCTION

In biology, the names of taxonomic groups (taxa) are defined by their association with nomenclatural types. For instance, in botany, the type might be plant specimens stored in a herbarium or a detailed illustration^1^. A plant with anatomical features similar enough to the type of a species to be classified in the same species must be given the same species name. Likewise, a plant whose features are judged sufficiently different from the type must be given a different species name. This system creates well‐defined, permanent names for species and other taxa. For Bacteria and Archaea, the International Code of Nomenclature of Prokaryotes (ICNP) requires that the nomenclatural types are strains that must be deposited in two international culture collections in at least two different countries, and such type strains must be available without restriction^2^. While effective for many prokaryotes, the ICNP does not allow for the creation of permanent names for uncultured and fastidious prokaryotes that cannot be deposited in culture collections. The SeqCode is a new code of nomenclature in which genome sequences are the nomenclatural types for the names of species^3^. While generally similar to the ICNP in structure and rules of priority, it does not require deposition of strains in international culture collections. Thus, it allows for the creation of permanent names for uncultured and fastidious prokaryotes. The start date of the SeqCode was January 1, 2022, and the online SeqCode Registry (https://seqco.de/) was created to ensure the valid publication of names under the SeqCode.

There are a number of compelling reasons to choose genome sequences as types for the SeqCode. Some are practical. Genome sequences avoid many of the disadvantages of strains as types^4^. Strains are expensive to maintain and difficult to share. Strains are genetically unstable^5^, in part because the selective forces in the laboratory differ from those in the natural habitat. Strains can also be lost. The Approved Lists^6^, which were completed in 1980, documented all the type strains for species known at the time. Although compiled just over 40 years ago, type strains have already been lost^7^. Thus, there is good reason to doubt the permanence and stability of type strains for centuries and even more extended periods of time. Moreover, the ICNP prohibits renaming taxa in the absence of strains deposited in two public culture collections, so the names of some species do not reflect the best scientific evidence for their classifications because they are classified in the wrong genera and cannot be renamed without deposition of either a neotype strain or a type strain in a second culture collection. Lastly, upon the application of molecular techniques to classification and systematics, especially sequencing of the 16S rRNA genes and later genome sequencing, it was discovered that comparison of strains by classical polyphasic taxonomy produced large errors, both in classifying distantly related species as sister taxa and failing to recognize synonyms^8^, ^9^, ^10^. In contrast, genome sequences have proven to be extremely reliable markers for speciation and taxonomy^11^, ^12^ and can be expected to have the long‐term archival permanence that preserved strains lack. Because of this, it is now well accepted that the description of new species should be accompanied by genome sequences^13^. Once all the genome sequences of the type strains are known, there is little practical reason not to consider the sequences themselves as types rather than the strains.

Extensive molecular ecological studies have revealed that most of the prokaryotes have never been—and may never be—cultured^14^, ^15^, ^16^. First, sequencing of 16S rRNA genes directly from environmental DNA demonstrated that the diversity of prokaryotes in nature is very different from that which is in culture. However, while the 16S rRNA gene sequence demonstrated that uncultured prokaryotes were enormously diverse, the so‐called “microbial dark matter”, it failed to provide insight into their functional properties. This problem was solved by the development of the technology to assemble and study metagenome‐assembled genomes (MAGs) and single‐cell amplified genomes (SAGs), where complete or nearly complete genome sequences could be obtained from environmental DNA^17^, ^18^, ^19^. For instance, direct sampling of DNA from many biomes has identified MAGs representing >160 prokaryotic phyla while only about 40 phyla are represented by cultures deposited in culture collections and named under the ICNP^17^. Phyla are the deepest evolutionary groups commonly recognized in prokaryotic systematics. They represent ancient lineages and differ greatly in their growth properties, cell structures, and overall metabolism. The observation that only approximately one‐quarter of the phyla have cultured representatives implies that there is an enormous gap in our understanding of the breadth of prokaryote diversity.

Similarly, although there are only general estimates of the total number of prokaryote species, the best guess is that no more than 0.2% have ever been cultured and named under the ICNP^15^. Even in a well‐studied microbiome like the human gut, many of the strains identified by metagenomic sequencing cannot be classified into known species or even known genera. In one study, 696 strains of prokaryotes were identified. Of these, only 460 could be assigned to a genus described under the ICNP. Likewise, only 321 could be assigned to species described under the ICNP^20^. This example illustrates that, even in the best‐studied microbiome from the human body, a large fraction of the prokaryotes remains undescribed and, thus, unnamed. The situation is like a city with 10 million people where only 20,000 people have proper names. Even though some of the unnamed millions have unusual and interesting talents or skills, they are excluded from being properly named by antiquated rules and instead are labeled with apparently random tags and numbers. How would we communicate with the people of such a city? Surely, we would want all these people to gain proper names. Now imagine 10 million prokaryotic species, where only 20,000 have proper names. By using genome sequences as types for uncultured taxa, the SeqCode makes it possible to provide proper names for a much larger portion of the prokaryotic species known to exist and, in doing so, will greatly improve communication among microbiologists.

Because genomes of most type strains have been sequenced^21^, ^22^, ^23^, the SeqCode will enable the creation of unified taxonomies of cultured and uncultured taxa as can be exemplified by the Genome Taxonomy Database^24^. Not only can these taxonomies identify closely related cultured and uncultured taxa, but they can also use uniform ranks for both groups. Thus, they can create a unified framework for the entirety of prokaryotic life. As an example of a unified taxonomy for closely related cultured and uncultured taxa, a recent paper^25^ describes the coordinated study of 794 pure cultures of the genus *Salinibacter* and *Salinibacter* MAGs from the same samples. One species was named under the ICNP upon deposition of the type strain in two culture collections. Because of difficulties in the deposition of a second type strain, the genome sequence of the strain was designated as the type, and the species was named under the SeqCode. Lastly, two MAGs were designated as the types for two additional species that remained uncultured, and these two species were named under the SeqCode.

## THE MODERN DESCRIPTION OF PROKARYOTIC LIFE

Since the first prokaryotic genome sequence was obtained less than 30 years ago^26^, genome sequences have become key to the modern description of prokaryotes. By identifying the presence and absence and context of genes in an organism, they provide access to a wealth of data on the biochemistry, molecular biology, and function of specific genes for use in deciphering the properties of an organism. They potentially inform us of key lifestyle features, such as modes of reproduction, cell envelope structure, differentiation, metabolism, stress responses, motility, and chemotaxis. Further, comparative genomics precisely defines the relatedness between organisms and enables the association of specific genes to phenotypes. Just as the genome sequence provides information about a taxon, the reverse is also true. Molecular, physiological, and ecological data about the taxon also inform the interpretation of genome sequences.

**Figure 1:**
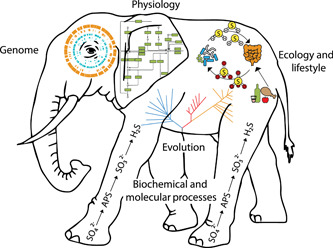
The name–elephant. Precise scientific names enable the unification of information from disparate disciplines to create a single, multifaceted vision of the taxon. Without names, the physiologist sees only the physiology, the ecologist sees only the ecology, and the molecular biologist sees only the molecular processes. A common vocabulary comprised of precise and permanent names is necessary for different disciplines to communicate effectively.

The result is a modern description of prokaryotic life comprised of multiple types of information generated from disparate disciplines, including the biogeochemistry of Earth's elemental cycles; the ecological interactions between microbes, other organisms, and the environment; the distribution of individual taxa in various habitats derived from surveys of 16S rRNA genes, MAGs, and SAGs; the lifestyle and physiology of individual microbes; the molecular processes defining metabolism, growth, and reproduction; and the evolution of organisms and the processes that define them. Genome sequencing and comparative genome analyses are key methodologies bringing these different types of information together. However, scientifically precise names are another essential component. Without names to facilitate communication among them, each discipline remains focused on only one aspect of an organism. In this regard, the disciplines are like the blind men and the elephant^27^. In this ancient parable, a group of blind men each try to ‘see’ an elephant by touching different parts. Because no blind man can touch the entire elephant, each has a different opinion about the elephant's nature depending upon the part they touch. Just as sight would allow one of the men to see the elephant in its entirety, a precise name allows investigators in disparate disciplines to bring together knowledge from many fields to create a unified vision of an entire organism (Figure 1).

As an example of how the correct name connects information on the distribution, physiology and metabolism, evolution, and ecological role, consider the genus *Desulfovibrio*. This genus of sulfate‐reducing bacteria is common in fresh, brackish, and salt water, but is also present in the intestinal tracts and feces of animals^28^. At one time, more than 70 phenotypically similar species were included in this genus. However, phylogenetic analyses of the 16S rRNA gene and whole genome sequences led to the reclassification of most of the species into other genera, leaving fewer than 10 closely related species in the genus^28^, ^29^. Of these, the type strain of *Desulfovibrio piger* was isolated from human feces^30^. Strains of *D. piger* possess 16S rRNA genes that are nearly identical in sequence, and genes with nearly the same sequence can be identified in human feces and colonic mucosal biopsies. Moreover, the closely related species *D. desulfuricans* and *D. legallii* ^29^ can also be readily isolated from human feces^31^. Even though their growth properties are similar to many other sulfate‐reducing bacteria in vitro, their genomic composition, distribution, and ecological roles differentiate them from those other bacteria. The presence of these bacteria in feces is of interest because they can grow by the anaerobic respiration of sulfate, producing hydrogen sulfide, which is toxic. Therefore, these sulfate‐reducing bacteria likely produce sulfide in the large intestine and might contribute toward intestinal diseases, including ulcerative colitis^32^ and several types of cancer^33^. Similarly, based on their genome content and biochemical studies, there is a firm understanding of the enzymology of their anaerobic respiration^28^. Most importantly, precise naming enables forming connections between ecological data, patient health, and physiological and molecular data and recognition of a group of bacteria potentially important to human health.

Uniting the nomenclature of cultured and uncultured prokaryotes also offers additional understanding, and knowledge of the uncultured can provide tremendous insights into the cultured. For instance, bacteria related to the genus *Chlamydia* are obligate intracellular parasites of eukaryotes^34^. *Chlamydia trachomatis* is a human pathogen and leading cause of a common sexually transmitted disease and blindness that was first ascribed to that species over a hundred years ago. Fifty years ago, it was thought of as an anomaly due to its obligately intracellular parasitism, unusual dimorphic life cycle, unusual cell wall, and other features^35^. Although humans are the only host of *C. trachomatis*, related uncultured species are widespread in other eukaryote hosts including protists and arthropods. Surveys of metagenomic databases suggest that the group contains at least 181 families and possibly over a thousand genera^36^. Moreover, the group is probably very ancient, having evolved over 700 million years ago^37^. Thus, a pathogen once largely considered to be anomalous is in fact representative of a major evolutionary lineage of prokaryotes. Since most of the species in the phylum *Chlamydiota* are either unculturable or cannot be deposited in culture collections because they are grown in protists, they have been given *Candidatus* names (see below). However, a panel of experts on the taxonomy of *Chlamydiota* recently recommended that names be registered in the SeqCode to ensure their stability and permanence^38^. Thus, the SeqCode may prove to be a valuable tool for creating a stable nomenclature and unraveling this group's evolution.

Evidence from studies of both uncultured and cultured taxa can provide insights into some of the central problems in biology. Since the discovery of Archaea as a separate domain from Bacteria more than 30 years ago by phylogenetic analysis of the 16S rRNA^39^, they have been found to be widely distributed in various Earth environments and to play essential roles in elemental cycling. However, the Archaea remain largely mysterious as the majority are uncultivated and have no cultured close relatives. Thus, MAGs and SAGs provide access to their genomes and enable inferring key genes for specific functions, metabolism, and phylogenetic relationships^40^, ^41^, ^42^. Analyses of MAGs have provided strong evidence that members of the Asgard lineage of Archaea may be the closest prokaryotic ancestor to the “nuclear” component of the Eukarya^43^, ^44^. Although this conclusion is still an area of active investigation^45^, ^46^, cultivation of a member of this lineage has led to the generation of the entangle–engulf–endogenize model for the origin of the eukaryotes and promises to yield additional insights into the nature of the earliest eukaryotes^47^.

**Figure 2:**
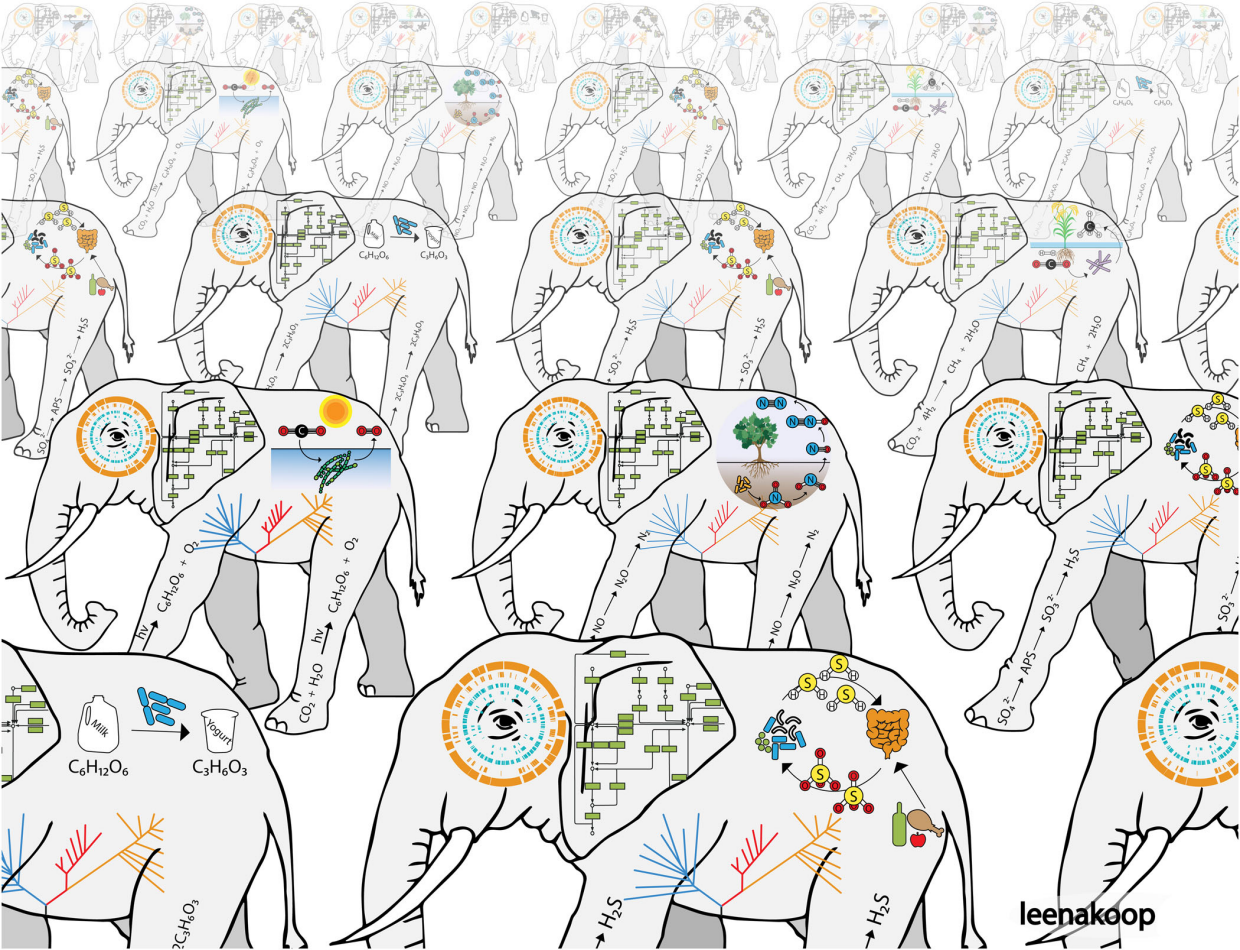
A herd of name–elephants. In practice, understanding evolutionary processes and the relatedness between species allows knowledge of one species to inform us about many different species. For example, functional characterization of protein X encoded by gene Y allows the simultaneous inference of the molecular function for orthologous sequences in all of life. Within a unified taxonomy for cultured and uncultured prokaryotes, knowledge from many disciplines and many species can be brought together for increased understanding and problem‐solving.

It must be recognized that most genomes are incompletely annotated, and laboratory experiments are necessary to inform the annotation process. Not only are the functions of many genes not known, but their functions in different genomic contexts and environmental conditions remain poorly understood. For instance, even after several decades, annotation of the genome of *Escherichia coli*, one of the best‐studied bacteria, is still incomplete^48^, ^49^. Nevertheless, the process of annotation is clearly understood and limited only by the investment of time and energy. Moreover, there have been great improvements in methods during the last decade, and we can expect that annotations will vastly improve in the future. Importantly, functional annotation of one gene in a specific genome contributes to the annotation of all genomes with that gene^50^. In a very real sense annotation is not performed on single species but, in fact, is performed on all of prokaryotic life together (Figure 2). Interpreting genome sequences relies heavily on molecular and biochemical studies of pure cultures. These insights can then be readily applied to the genome sequences of uncultured prokaryotes, providing both clues to the organisms' phenotypes as well as strategies for their isolation^19^, ^41^. The association of culture metadata with genome sequences provides another avenue of exploration^51^, and it may be possible to identify the genetic basis for specific phenotypic traits.

An integrated description of prokaryotic life includes an understanding of the evolutionary processes and the relationships between species. Such an approach can identify knowledge gaps and support educated hypotheses of the properties of organisms from what is known about their relatives. By identifying gaps in our knowledge, it guides research to complete the description. Consider the metaphor of a library where every book is a prokaryotic species. If our goal is to learn the complete contents of the library, one strategy is to read every book. Given that there might be 10 million books in the library, this is an inefficient approach. Alternatively, if the books are arranged in a logical order where some of the contents are shared and some are unique, we could learn the complete contents of the library by understanding the order of the books, discovering which parts are shared, and then focusing only on reading the unique parts of each book. The study of evolution informs us of the logical order, but it cannot possibly be complete if it only includes cultured organisms.

While the modern description of prokaryotic life is of theoretical interest, it also has great practical value. It enables efficient and thorough biomining of prokaryotic diversity for genes and organisms for biotechnological applications. For instance, antibiotics, restriction endonucleases, thermostable DNA polymerases, and CRISPRs were discovered during studies of prokaryotic biology, all of which have had enormous impacts on modern medicine and biotechnology. Surely other biological “gadgets” are waiting to be discovered. The modern description enables the exhaustive identification and characterization of bacterial pathogens, over 1500 of which have been identified in humans before 2021^52^. Prokaryotes also catalyze many of the critical steps in the Earth's biogeochemical cycles. The modern description will improve our understanding of the biogeochemical processes that control the climate and responses to global warming. Prokaryotes play prominent roles in soil fertility and animal husbandry, and the modern description will have direct applications in agriculture. Likewise, the modern description will make possible the examination of fundamental questions, such as do prokaryotic species go extinct and could their extinctions lead to the loss of essential ecological services? In fact, prokaryotes impact almost every aspect of human life, and a thorough understanding of their biology will have a profound impact on our lives.

## WHAT IS THE SeqCode?

By providing a precise and stable nomenclature, the SeqCode is an important component of the modern description of prokaryotes. Because genome sequences are the nomenclatural types for the names of species, the SeqCode does not require deposition of type strains in international culture collections and allows for the creation of permanent names for uncultured and fastidious prokaryotes^3^, ^53^. Here, fastidious prokaryotes refer to organisms that have been cultured but whose growth requirements are so difficult or specialized that they cannot be efficiently maintained in culture collections. The SeqCode can also be used to name cultured organisms when the genomes are sequenced, allowing the naming of species discovered in countries where the international exchange of strains is prohibited by the national implementation of the Nagoya Protocol^54^. Because the SeqCode is compatible with the ICNP in many aspects, it is now possible to create unified taxonomies of cultured and uncultured organisms. In fact, by recognizing the names validly published under the ICNP, the SeqCode is already a step in that direction. Lastly, it sets data standards for type genome sequences and encourages good practices in naming, metadata collection, and data sharing. Importantly, the SeqCode requires the deposition of raw sequence data from which type genomes are derived so the data can be reanalyzed by other investigators–a practice rarely implemented before the SeqCode.

SeqCode names differ fundamentally from *Candidatus* names, which were originally introduced to name uncultured taxa under the ICNP^55^, ^56^. At the time the *Candidatus* status was proposed, sequencing of 16S rRNA genes from environmental DNA had revealed the abundance of uncultivated taxa in the biosphere, but the technology to generate whole genome sequences from MAGs and SAGs was not available. More recently, *Candidatus* names have also been widely used to name fastidious prokaryotes, which cannot be maintained by culture collections. Most importantly, *Candidatus* names are outside the formal rules of the ICNP^2^. As the name implies, they are provisional and lack standing in nomenclature as well as priority. In the context of systematics, priority refers to the permanent association of a single name to a taxon and is a fundamental concept in most codes of nomenclature^1^, ^2^, ^3^, ^57^. Because *Candidatus* names are not protected by rules of priority, it is not unusual for different investigators to assign different *Candidatus* or informal names for the same taxon. Upon isolation of a culture from a *Candidatus* taxon, it is possible to create and validate an entirely new name under the ICNP. The potential for redundancy and lack of permanence are fundamental flaws requiring frequent curation and preventing the efficient use of *Candidatus* names in expanding modern databases. Lastly, because many *Candidatus* names lack nomenclatural types, their meanings are inherently ambiguous and lack precision.

As an extreme but common example of the ambiguity of *Candidatus* and informal names, it has become common to assign names to higher ranks, particularly phyla, without designating any lower ranks or making any effort to circumscribe the taxon. Thus, the poor structure of the ICNP *Candidatus* nomenclature promotes a chaotic situation in prokaryotic biology^58^ that has ironically been used to argue against the development of a well‐ordered system to name uncultured prokaryotes^59^. It is important to ask: *Why should we adhere to a well‐organized structure for naming and cataloging cultured prokaryotes and then abandon that structure when studying uncultured prokaryotes?* In summary, although *Candidatus* is described in an appendix of the ICNP, we recommend abandoning its use in favor of the stronger structure and data standards implemented under the SeqCode.

In addition to providing its own framework for the valid publication of names, the SeqCode also recognizes all names validly published under the ICNP before January 1, 2022. After that date, SeqCode names compete with ICNP names for priority. This means that a taxon first named under the ICNP cannot be renamed under the SeqCode even though the type of one is a strain and of the other is a genome sequence. Although the ICNP does not formally recognize the SeqCode (or names formed under it)^60^, if a taxon is first named under the SeqCode and subsequently renamed under the ICNP, the SeqCode name will have priority over the ICNP name. Otherwise, the rules of the SeqCode and ICNP are very similar with the intention that the two codes will someday be united, creating a unified nomenclature for all prokaryotes.

## USING THE SEQCODE REGISTRY

**Figure 3:**
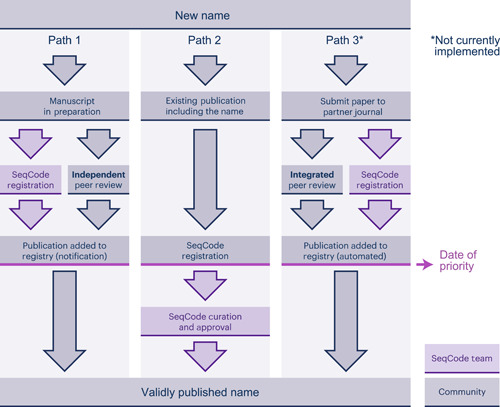
Validation process of names under the SeqCode (modified from Hedlund et al.^3^). Currently, two mechanisms exist with a third planned for the future. In Path 1, the name and metadata are entered into the SeqCode Registry concurrently with the preparation of the effective publication. Path 2 is for names that are already published, such as *Candidatus* names. After review by the SeqCode curators, the names can be registered, marking the date of priority. Path 3 could be developed in partnership with one or more journals and would involve simultaneous peer review and Registry curator review as an integrated path to the validation of proposed names.

The SeqCode Registry is an online resource for registration and validation of names under the SeqCode. The Registry provides a list of names validated under the SeqCode similar in purpose to the Validation Lists of the *International Journal of Systematic and Evolutionary Microbiology* (*IJSEM*). Table 1 and Figure 3 outline the steps required for entry of a new name into the Registry, which can occur before or during peer review of a manuscript (Path 1) or after the effective publication is published (Path 2). This structure allows the SeqCode to accommodate taxonomic names proposed in existing publications through Path 2 but also provides support for the research community on etymology, orthography, and data standards before publication through Path 1. In addition, well‐justified exceptions for some of the requirements can be requested and approved by the curators and SeqCode Committee during registration. In the future, Path 1 is strongly recommended because the curators can support and encourage best practices and ensure that published names can be validated, thus promoting consistency and stability between the Registry and the greater body of literature. In brief, the process entails registration of a name and required data fields by the contributor, submission of the entry in the form of a register list for evaluation by curators, evaluation and endorsement of the name and genome quality by Registry curators, and effective publication of a manuscript on the taxon. Validation and the date of priority are then established by the authors' completion of required fields, typically the entry of the digital objective identifier (DOI) of the effective publication in the Registry.

**Table 1:** Naming new taxa under the SeqCode.

Steps for naming new taxa under the SeqCode	Yes	No
1. Does the type genome meet the minimum standards? (>90% complete and <5% contamination for MAGs and SAGs, and >10‐fold coverage for isolate genomes)	Proceed	Consult with curators if there is a reason for low completion or contamination. For instance, the completeness of small genomes is frequently underestimated, and this criterion can be misleading. Similarly, some polyploid organisms contain multiple genomes with different sequences and appear to be contaminated when, in fact, they are not^37^. In these cases, the genomes could still serve as types even though they do not appear to meet the standards.
2. Are genomic assembly and sequence reads available in an INSDC database?	Proceed	Deposit assembly in an INSDC database and sequence reads in a Sequence Read Archive or Nucleotide Archive and ensure data are released.
3. Is the ANI of the type genome <95% to all SeqCode type genomes and genomes of all type strains of species named under the ICNP?	Proceed	Consult with curators and provide the rationale for a new species in the effective publication.
4. Is there a species named in the ICNP with a similar 16S rRNA sequence whose genome has not been sequenced?	Sequence the genome of this species and return to step 3.	Proceed
5. Does the new species belong to a genus currently named in either the ICNP or the SeqCode?	Choose a species epithet not currently used in this genus. In the effective publication, the name is designated “sp. nov.”	Name the new genus. In the effective publication, the name is designated “gen. nov., sp. nov.,” and register the new genus name in the SeqCode Registry for simultaneous evaluation and validation.
6. Does the genus belong to a currently named family?	Indicate the family in the effective publication and the SeqCode Registry.	Name the new family with your new genus as a type, designate it as “fam. nov.” in the effective publication, and register the new family name in the SeqCode Registry for simultaneous evaluation and validation. If you are unsure of the taxonomic placement, indicate the taxon as *incertae sedis*.
7. Does the family belong to a currently named order?	Indicate the order in the effective publication and the SeqCode Registry.	Name the new order with your new genus as type, designate it as “ord. nov.” in the effective publication, and register the new order name in the SeqCode Registry for simultaneous evaluation and validation. If you are unsure of the taxonomic placement, indicate the taxon as *incertae sedis*.
8. Does the order belong to a currently named class?	Indicate the class in the effective publication and the SeqCode Registry.	Name the new class with your new genus as type, designate it as “class. nov.” in the effective publication, and register the new class name in the SeqCode Registry for simultaneous evaluation and validation. If you are unsure of the taxonomic placement, indicate the taxon as *incertae sedis*.
9. Does the class belong to a currently named phylum?	Indicate the phylum in the effective publication and the SeqCode Registry.	Name the new phylum with your new genus as type, designate it as “phyl. nov.” in the effective publication, and register the new phylum name in the SeqCode Registry for simultaneous evaluation and validation. If you are unsure of the taxonomic placement, indicate the taxon as *incertae sedis*.
10. Have you submitted your names on a Registry List for evaluation?	Curators will evaluate the name and assess the quality of the genome/s to serve as type and endorse names or return them to the submitter to amend.	Confirm all necessary information is filled out in the SeqCode Registry, add all names appearing in the same effective publication on a single Registry List, and submit the Registry List for evaluation.

The Registry requires deposition of the type genome assembly in an INSDC database and the raw sequence data in a Sequence Read Archive (SRA) entry with associated metadata in a BioSample. The rationale for using INSDC databases is that they provide a permanent repository for the sequence data, enable the association of metadata with the sequence in standardized formats, and enable automatic checking of the sequence quality by the SeqCode curators. The rationale for deposition in the SRA is that it enables reassembly of the genome sequence as better methods are developed as well as testing for artifacts. Although not required, the addition of metadata to the assembly is expected to have great utility in subsequent meta‐analyses, especially as the size of the Registry grows. Thus, another major objective of the Registry is to make metadata of named taxa freely available online for querying and analysis, a significant departure from the previous practices that required painstaking searching of archived journal articles to recover this important information. An effective publication, which includes the proposed name of the taxon and preferably a description and circumscription of the taxon, is also required and can be in any peer‐reviewed journal. Validation in the Registry for endorsed names is concluded upon entry of the digital object identifier or DOI of the effective publication, a step called Notification.

## STANDARDS FOR GENOME SEQUENCES USED AS NOMENCLATURAL TYPES

The recovery of the genomes of uncultured taxa from environmental samples, in the form of MAGs or SAGs, rarely provides complete genome sequences; typically, an incomplete draft genome is obtained^61^. Therefore, a key element of taxon descriptions using the SeqCode is the deposition of high‐quality genomes into the public databases to serve as nomenclatural types. The administrative groups of the SeqCode (see below) have come up with the first set of standards that should be met by these type genome sequences^3^. These standards are outlined in Table 1 and are inspired by the minimum standards for an isolate genome, a MAG sequence, and a SAG sequence proposed by the Genomics Standard Consortium^61^. The standards (e.g., >90% completeness and <5% contamination for MAGs and SAGs) may appear too stringent compared to the current practice in reporting MAGs/SAGs in the literature. However, high quality is necessary to ensure precise and stable phylogenetic placement of the taxon and inference of its functional properties^3^, ^62^. Although not a requirement, species descriptions should be based on two or more MAGs or SAGs from more than one sample, and descriptions based upon a single MAG or SAG recovered from a single sample should be avoided. Finding similar genomes in more than one sample adds valuable ecological data and helps with the identification of chimeric genomes (e.g., obtaining closely related genomes from two or more samples is evidence that the genomes are free of artifacts). Further, should a type genome prove to be chimeric, and thus lead to misleading phylogenetic and/or functional inferences, it is possible to replace such sequences with new, improved sequences, or a neo‐type, similar to the strain replacement when the type strain is lost under ICNP. The SeqCode also allows genome sequences of lower quality to be used as types if the authors can justify why obtaining higher quality genomes is not technically feasible and provide strong support for the phylogenetic placement of the organisms to be named (see Table 1 for details). The administration of the SeqCode includes a plan to update the genome standards based on improvements in wet‐lab (e.g., long‐read sequencing) and bioinformatics methodologies (e.g., new genome binning and quality assessment tools) that emerge in the future and can provide more complete genome sequences from uncultivated taxa (see below for more details). Using genome sequences as types has also been criticized as failing to represent the dynamic properties of an organism and being error prone^63^, ^64^. However, the advantages of using the genome sequences for this purpose outnumber the disadvantages, as has been argued elsewhere^65^, ^66^.

## ADMINISTRATION OF THE SeqCode

The SeqCode was published on September 19, 2022^3^, and its administrative structure was formed on January 1, 2023, to oversee its operation and provide mechanisms to institute changes (SeqCode Statutues 1.0.pdf [isme-microbes.org]). The SeqCode Committee is comprised of the SeqCode Community and five administrative components. Anyone with an interest in the systematics of prokaryotes, demonstrable through publications or employment, as well as members of the International Society of Microbial Ecology (ISME) are welcome—indeed, encouraged—to join the SeqCode Community at this website (SeqCode Community Signup [seqco.de/join]). The administrative components include the Executive Board, which is entrusted with the overall management of the SeqCode Committee. It is comprised of its officers and the Chairs of the other administrative groups; both officers and Chairs are elected by the SeqCode Community. The administrative groups include: (i) the Legislative Commission, which is responsible for overseeing the amendment of the SeqCode when the need arises; (ii) the Reconciliation Commission, which is responsible for the interpretation of the SeqCode and which should be contacted when the interpretation of the SeqCode is ambiguous or for consideration of names contrary to the code; (iii) the Registry and Nomenclature Working Group, which is responsible for the development and operation of the SeqCode Registry and which can also provide nomenclatural advice for creating taxon names; and (iv) the Standards Working Group, which is responsible for monitoring technical development in generating sequence data and providing recommendations for the minimal data standards. The officers and members of these administrative groups can be found on the SeqCode Committee page of the Registry (http://seqco.de/committee). The SeqCode is a “living” document, and the Statutes provide procedures to both interpret and introduce changes as technology and experience dictate. Questions of interpretation should be directed to the Reconciliation Commission. Although the SeqCode was purposefully written to closely follow many of the tenets of the ICNP, its implementation is likely to bring to light areas where the code can be improved. Hence, proposals to change the SeqCode should be directed to the Legislative Commission following publication in a peer‐reviewed journal article explaining the rationale for the proposed changes. Moreover, as the technology used in systematics is expected to improve, the SeqCode will need to permit the implementation of the best methodology as it becomes available. The Standards Working Group is responsible for ensuring that the best technical standards are employed.

While the Registry is currently operational, a paramount goal now is its further development. The intention is to make the Registry user‐friendly and self‐sustaining, recognizing that nomenclature is the tail and should not wag the dog of systematics. The Registry should provide a service to facilitate naming without imposing artificial barriers. Ideally, names will be validated by the creators with minimal input from a curator, and manual curation should be reduced as much as possible with automatic checks of sequence data and name etymology. While the current system is already semi‐automated, further automation in the validation processes under the supervision of expert Latinists and genome biologists will reduce operational costs and labor, while simultaneously increasing reproducibility and transparency. These goals are readily achievable with the available data‐processing technology and only await the allocation of resources. For example, the system already features algorithms and heuristics from Latin grammar that have significantly reduced the curation time and allowed users to self‐evaluate the quality of their proposed names. However, automated methods to deal with compound words, methods that require access to a Latin dictionary, or methods that require inferring declensions and grammatical cases could all be implemented if additional resources were available. Similarly, additional developments and implementations of genomic methods, notably on the “exceptions” among prokaryotes (e.g., streamlined genomes and polyploid genomes) will further improve the quality of the curation, simplify registration, and increase the utility of the SeqCode Registry as a digital repository of reference material. As more taxa are discovered, bringing to light novel taxonomic groups in need of a stable nomenclature, these developments are necessary to keep pace with the rapid growth of the new prokaryotic biology.

During the writing of the SeqCode, it was debated whether or not names should be Latin words. There are plausible arguments for allowing names to be formed in any language and even arbitrary syllables^67^. Presumably, the tradition of using Latin names remains from a time when Latin was a common component of most scientists' education. It is less justifiable in the modern international scientific community. Names formed from any language would be easier to create and still retain their utility. The major rationale for using Latin names in the SeqCode is to retain compatibility with the ICNP and facilitate the eventual merger of the two codes. Should the ICNP relax its rules about the formation of names in Latin, the SeqCode should follow suit. Lastly, the “Great Automatic Nomenclator” (GAN) promised a technological solution to difficulties in creating Latin names at scale and was developed at about the same time as the SeqCode^68^. GAN can generate large numbers of names from a small number of Latin roots. Thus, the implementation of GAN in the SeqCode Registry is a high priority. Currently, users are encouraged to use the version of GAN implemented in the Protologger tool (http://www.protologger.de/ ^19^).

In the meantime, a brief primer on forming Latin names for SeqCode taxa is presented in Box 1. In due course, it seems likely that AI technologies will soon emerge that will facilitate the easy formation of grammatically correct Latin names. When this happens, it could well be implemented in the Registry.

Box 1 **Some simple rules for creating Latin names for taxa under the SeqCode**

1. Rules for all names

a. The scientific names of all taxa must be treated as Latin and spelled only with the Latin alphabet. A species name is a binary combination of a genus name and a specific epithet; names of taxa above the rank of species are single words. Typographical signs, numbers, and additional characters cannot be used.
b. The phrase “treated as Latin” means that, while a name can be derived from any source, it should be consistent with the rules of Latin grammar and contains an ending consistent with Latin. In general, use a word other than Latin only when there is no Latin word with the same meaning.
c. All names comprise only the 26 letters of the ISO basic Latin alphabet. Diacritic signs are not to be used.
d. Any name or epithet should be written in conformity with the spelling of the word from which it is derived and in accordance with the rules of Latin grammar.
e. The derivation (etymology) of a new name is given wherein one or more distinguishable roots are identified. Roots can originate from any language in use or extinct and can be formed from acronyms.
f. See Recommendations 9 and 10 of the SeqCode for further suggestions about good practices.

2. Forming a species name [for detailed guidance see Appendix 9 of Ref.^69^]
  a. Species names are binomial consisting of genus names and specific epithet. If the genus is not named, name it first. When you have a genus name, proceed.
  b. A species epithet must be related to the genus name in one of three ways.
    i. As an adjective. In this case, the gender of the adjective must match the gender of the genus name. Genders include masculine, feminine, and neuter. An example would be an epithet based on a location. In this case, if the genus is masculine or feminine, add the ending ‐*ensis* to the name of the place. If the genus is neuter, add the ending ‐*ense*. If the name of the place ends in ‐a or ‐e, drop these letters before adding ‐*ensis* or *‐ense*. However, there is an exception if Latin already has an adjective for the place. In that case, use the Latin adjective.
    ii. As a noun in apposition in the nominative case. The nominative case is the form of a noun that is used as the subject of a verb. Not all languages distinguish the case of nouns. [see 3.c below]
    iii. As a noun in the genitive case. In this context, the genitive case is used to indicate that the noun possesses or denotes an attribute of the genus. [see 3.c below]
  c. Species epithets can be generated from single or compound words. The formation of compound words is discussed below in 3.d.
  d. See Trüper and De'Clari^70^ for more discussion on species names.
3. Forming a genus name [for detailed guidance see Appendix 9 of Ref.^69^]
  a. The name of a genus is a noun or adjective used as a noun, in the singular number, and written with an initial capital letter.
  b. Decide what kind of name you want to create. Options are endless but here are some common approaches:
    i. Based on the personal name [see Appendix 9D of Ref.^69^]
    ii. Based on the organism's features
    iii. Based on location [see Appendix 9E of Ref.^69^]
    iv. Based on the host [see Appendix 9F of Ref.^69^]
  c. Use dictionaries to identify Latin equivalents for the chosen word/s. The dictionaries will usually provide the nominative singular followed by the genitive singular. If your name consists of one word, it should be a noun in the singular case; i.e., the nominative singular.
  d. If the names consist of more than one word, identify the stem of each word. [see: How To Find the Stem of Any Latin Noun (Easily!)—Books “n” Backpacks (booksnbackpacks.com)] Do any of the words start with a vowel (a, e, i, o, or u)? If yes, they can be appended immediately following the stem of another word. If no, use the connecting vowel “i” to join the words. Although the SeqCode treats words from all languages as Latin, “o” as a connecting vowel may still be used for names with Greek roots.
  e. A genus name already in use in the botanical or zoological code cannot be used. Use a search engine to determine if your proposed name is already in use. Additionally, the SeqCode Registry also checks for names across codes of nomenclature, so you can confirm the uniqueness of your proposed name simply by registering it in the system.
4. Forming names of higher taxa.
  a. Identify the stem of the name of the type genus. If unsure, this process can be facilitated by the SeqCode Registry, which will provide the inferred stem of any registered genus in the Nomenclature section of the name. If the genus name is a compound word, consider only the last word. The other words and their connecting vowels are not changed.
  b. For family names, attach the suffix ‐*aceae* to the stem of the type genus name.
  c. For order names, attach the suffix ‐*ales* to the stem of the type genus name.
  d. For class names, attach the suffix ‐*ia* to the stem of the type genus name.
  e. For phylum names, attach the suffix ‐*ota* to the stem of the type genus name.

## RELATIONSHIP OF THE SeqCode TO THE ICNP

The SeqCode was formed with the intention that it can complement and then someday be merged with the ICNP. For that reason, names formed under the ICNP automatically obtain priority in the SeqCode unless they conflict with the priority of a SeqCode name. For instance, if a new species is described under the ICNP and the genome sequence of its type strain is judged to be sufficiently different from the type of any species named under the SeqCode to warrant classification in a new species, it should be considered a valid name under the SeqCode even if it is not entered into the SeqCode Registry. However, if the genome of the type strain is not sufficiently different, for instance, it might possess an average nucleotide identity (ANI) > 95% to the type of a species named under the SeqCode^71^ and lack any distinguishing characters to justify classification in a separate species, the name would violate the priority of the SeqCode name and be illegitimate under the SeqCode.

Because the SeqCode recognizes names validly published under the ICNP, it also permits adding subordinate taxa to higher taxa named under the ICNP. This is especially important given the binomial nomenclature of species, which includes both the genus name and species epithet. Thus, adding species to genera named under the ICNP is permitted under the SeqCode. Unfortunately, so far, the ICNP does not reciprocate this approach. Because it does not recognize names formed under the SeqCode, it is not possible to add species to genera named under the SeqCode following the rules of ICNP, and it is possible to name taxa under the ICNP that have already been named under the SeqCode. Therefore, we must rely upon the goodwill and common sense of investigators to avoid generating conflicts between the two codes of nomenclature. A possible solution to naming a species under the ICNP in a genus named under the SeqCode is to create an ICNP name identical to the SeqCode genus name with a unique species epithet. Although the ICNP and SeqCode genus names will differ in their types, defining publications, and priority, this will minimize any confusion regarding their meaning and have little effect on their use.

For higher taxa, the situation is somewhat different. Both the SeqCode and the ICNP require that the names of families and higher taxa are formed from the stem of the type genus name. However, the SeqCode requires that the type genus be the first validly published genus in the taxon while the ICNP allows any genus in the taxon to be the type. If the name of the first validly published genus was formed under the ICNP, it could be chosen to be the type of the higher taxa names formed under the ICNP. In that case, the SeqCode and ICNP names of the higher taxa will be identical. If another genus is chosen to be the type under the ICNP, the higher taxon name will be illegitimate under the SeqCode if it was validly published after January 1, 2022. However, if the first validly published genus was formed under the SeqCode, it cannot serve as the type of the ICNP names. As a consequence, names of the higher taxa formed under the ICNP will not be legitimate under the SeqCode. Confusion can then be minimized by creating a synonym to the SeqCode genus name in the ICNP as described above. Then the names will be the same although the types, defining publications, and priority will be different. While it remains to be seen to what extent this problem will occur, case studies will likely need to be reviewed so that workaround solutions can be identified. In the meantime, we suggest the formation of names of higher taxa under the SeqCode to avoid conflicts between the two nomenclatures.

The International Committee on Systematics of Prokaryotes and the SeqCode Commission are also encouraged to engage in dialogue with each other and the wider microbiology research community to explore permanent mechanisms for merging the ICNP and the SeqCode.

Lastly, naming taxa under the SeqCode does not and should not prevent efforts to isolate representatives of taxa and deposit strains in culture collections. In fact, naming important uncultivated taxa should promote efforts to isolate representatives of these taxa for their further study^3^. The availability of cultures in culture collections is of great value for the modern description of prokaryotes. Even when its genome is known, the current state of the art poorly predicts the properties of an organism, and laboratory experiments on living organisms are still necessary^63^, ^64^.

## NOMENCLATURE SHOULD BE PROACTIVE

Prokaryotic nomenclature underwent a major reform with the recognition that type strains were necessary to create a solid foundation for the nomenclature of prokaryotes. This realization led to the Approved Lists and requirements for type strains for new names formed under the ICNP^72^, ^73^. As a result of these reforms, well‐documented cultures were assembled in collections. Even though the rapid growth of DNA sequencing technology was probably not anticipated when these reforms were initiated, the availability of type strains enabled the extensive 16S rRNA gene and genomic sequencing of the last 40 years and a major paradigm shift in prokaryotic biology. In the same spirit, the SeqCode also strives to be proactive. Although it is difficult to anticipate what will develop from the unification of the nomenclature of cultured and uncultured prokaryotes, the SeqCode and modern technology will make it possible to address these future needs.
